# Enhanced Photovoltaic Properties of Y6 Derivatives with Asymmetric Terminal Groups: A Theoretical Insight

**DOI:** 10.3390/ijms241914753

**Published:** 2023-09-29

**Authors:** Yunjie Xiang, Zhijun Cao, Xiaolu Zhang, Zhuo Zou, Shaohui Zheng

**Affiliations:** 1 School of Materials and Energy, Southwest University, Chongqing 400715, China; hao1986kx88@swu.edu.cn (Y.X.); czj0814@email.swu.edu.cn (Z.C.); zxl207842@email.swu.edu.cn (X.Z.); 2 Chongqing Key Laboratory for Advanced Materials and Technologies of Clean Energies, Southwest University, Chongqing 400715, China; 3 School of Materials Science and Engineering, Suzhou University of Science and Technology, Suzhou 215009, China

**Keywords:** organic solar cell, asymmetric Y6 derivatives, terminal group, photovoltaic properties

## Abstract

Y6 derivatives with asymmetric terminal groups have attracted considerable attention in recent years. However, the effects of the asymmetric modification of terminal groups on the photovoltaic performance of Y6 derivatives are not well understood yet. Therefore, we designed a series of Y6-based acceptors with asymmetric terminal groups by endowing them with various electron-withdrawing abilities and different conjugated rings to conduct systematic research. The electron-withdrawing ability of the Y6-D1 terminal group (substituted by IC-2F and IC-2NO_2_ terminals) is strongest, followed by Y6 (substituted by two same IC-2F terminals), Y6-D2 (substituted by IC-2F and 2-(4-oxo-4,5-dihydro-6H-cyclopenta[b]thiophen-6-ylidene)malononitrile terminals), Y6-D4 (substituted by IC-2F and indene ring), and Y6-D3 (substituted by IC-2F and thiazole ring). Computed results show that A–A stacking is the main molecular packing mode of Y6 and four other asymmetric Y6 derivatives. The ratios of A–A stacking face-on configuration of Y6-D1, Y6-D2, Y6-D3, Y6-D4, and Y6 are 51.6%, 55.0%, 43.5%, 59.3%, and 62.4%, respectively. Except for Y6-D1 substituted by the IC-2F and IC-2NO_2_ (the strongest electron-withdrawing capacity) terminal groups, the other three asymmetric molecules are mainly electron-transporting and can therefore act as acceptors. The open-circuit voltages of organic solar cells (OSCs) based on Y6-D2, Y6-D3, and Y6-D4, except for Y6-D1, may be higher than those of OSCs based on the Y6 acceptor because of their higher energy levels of lowest unoccupied molecular orbital (LUMO). PM6/Y6-D3 and PM6/Y6-D4 have better light absorption properties than PM6/Y6 due to their higher total oscillator strength. These results indicate that Y6-D3 and Y6-D4 can be employed as good acceptors.

## 1. Introduction

Currently, the power conversion efficiency (PCE) of single-junction organic solar cells (OSCs) based on nonfullerene acceptor (NFA) Y6 derivatives has reached 19% [1]. Y6 derivatives can be classified into two categories based on their chemical structure: symmetric [2,3] and asymmetric molecules. Asymmetric Y6 derivatives generally have stronger binding energies and higher dipole moments than symmetric Y6 derivatives, increasing the interactions between molecules. By adopting an asymmetric strategy, the photoelectrical properties of Y6 derivatives can be flexibly changed; not only their orbital energy levels, absorption, molecular stacking, and compatibility with donors can be altered but also their crystallinity and charge transfer ability can be varied [4]. Based on these advantages, an asymmetric strategy can effectively help develop high-performance, nonfullerene OSCs.

Until now, the asymmetric modification of Y6 has mainly been categorized into three aspects: end groups, frameworks, and side-chain asymmetric modification. Due to a relatively high open-circuit voltage (V_OC_) and fill factor, OSCs based on Y6 derivatives with asymmetric terminal groups have the highest PCEs of these three kinds of asymmetric Y6 derivatives [4]. Therefore, Y6 derivatives with asymmetric terminal groups play a crucial role in OSCs, and it is of great significance to design new Y6 derivatives with asymmetric terminals to develop OSCs. In 2020, Chen et al. developed two novel asymmetric Y6 derivatives, BTP-S1 and BTP-S2, with two different conjugated end groups composed of halogenated indandione and 3-dicyanomethylene-1-indanone on a benzothiadiazole (BT)-based central fused-ring core [5]. The PCE of PM6:BTP-S2-based OSC is 16.37%, which is higher than that of OSCs based on PM6:Y6 (15.79%). A ternary device based on PM6:Y6:BTP-S2 exhibited an optimal PCE of 17.43%, which was one of the highest efficiencies for a single-junction OSC at that time. In 2021, Chen et al. reported asymmetric Y6 derivatives BTP-S8 and BTP-S9 with two terminals of 2-(5,6-dichloro-3-oxo-2,3-dihydro-1H-inden-1-ylidene) malononitrile (IC-2Cl) and 2-(6,7-difluoro-3-oxo-2,3-dihydro-1H-cyclopenta[b]naphthalen-1-ylidene) malononitrile (NC-2F) [6]. An OSC based on PM6:BTP-S9 achieved a PCE of 17.56% (certified: 17.4%), which is higher than that of an OSC based on PM6:BTP-S8 (17.33%). In 2022, Hai and co-workers studied an asymmetric Y6 derivative BTP-S, which consisted of two IC-2F groups and a vinylene π-bridge on either side of the aromatic core [7]. The device based on PBDB-T: BTP-S achieved a PCE of 12.56%. In recent years, Sun group has studied an asymmetric acceptor LC301 with electron-withdrawing 2-(6-oxo-5,6-dihydro-4H-cyclopenta[c] thiophen-4-ylidene) malononitrile and 2-(5,6-difluoro-3-oxo-2,3-dihydro-1H-inden-1-ylidene) malononitrile (IC-2F) end groups. Furthermore, a device using a polymer PM6 as the donor and asymmetric LC301 as the acceptor achieved a PCE of 17.2% [8,9]. These studies indicate the following problems that currently exist in asymmetrically modifying Y6 derivatives: (1) the asymmetric modification of terminal groups is insufficient, and (2) although OSCs based on the above asymmetric Y6 derivatives have achieved slightly higher PCEs, it is still unclear how the asymmetric modification of terminal groups affects the photovoltaic performance of Y6 derivatives.

**Figure 1:**
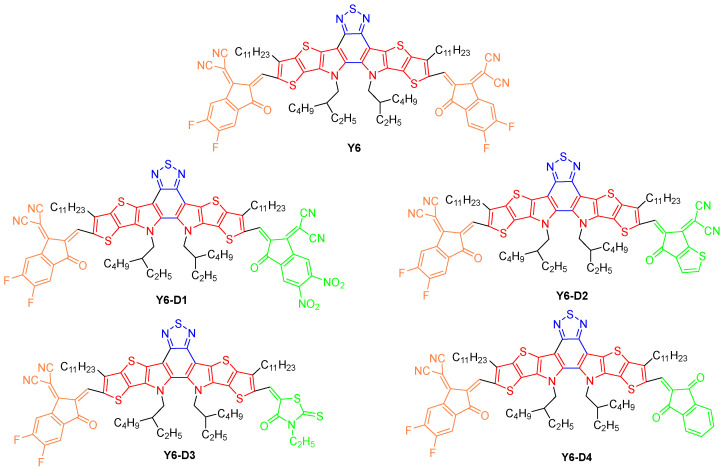
Chemical structure of newly designed Y6-D1, Y6-D2, Y6-D3, and Y6-D4 with an A_1_–D–A_2_–D–A_3_ structure, where A_3_ is replaced by different end groups (A_1_: yellow; D: red; A_2_: blue; A_3_: green).

Herein, we substitute one IC-2F terminal of symmetric Y6 by the groups with different electron-withdrawing ability and conjugated ring to design asymmetric Y6-D1 (substituted by IC-2F and IC-2NO_2_ terminals) (Figure 1), Y6-D2 (substituted by IC-2F and 2-(4-oxo-4,5-dihydro-6H-cyclopenta[b]thiophen-6-ylidene)malononitrile terminals), Y6-D3 (substituted by IC-2F and 3-ethyl-2-thioxothiazolidin-4-one terminals), and Y6-D4 (substituted by IC-2F and 1H-indene-1,3(2H)-dione terminals). IC-2F is named the A1 terminal group, and the electron-absorbing group on the other side is named the A3 terminal group. The terminal group of Y6-D1 exhibits the strongest electron-withdrawing ability, followed by Y6, Y6-D2, Y6-D4, and Y6-D3. Furthermore, the electrostatic potential (ESP), molecular packing, reorganization energy, charge carrier mobility, frontier molecular orbitals (FMOs), energy gap, and UV–Vis spectra are theoretically calculated for investigating the effects of modifications using asymmetric terminals on the photovoltaic performances of Y6 derivatives. Ultimately, potential asymmetric acceptors are screened out. This research provides a guideline for future research on modification using Y6 asymmetric terminal groups. 

## 2. Results and Discussion

### 2.1. ESP of PM6, Y6, and Asymmetric Y6 Derivatives

**Figure 2:**
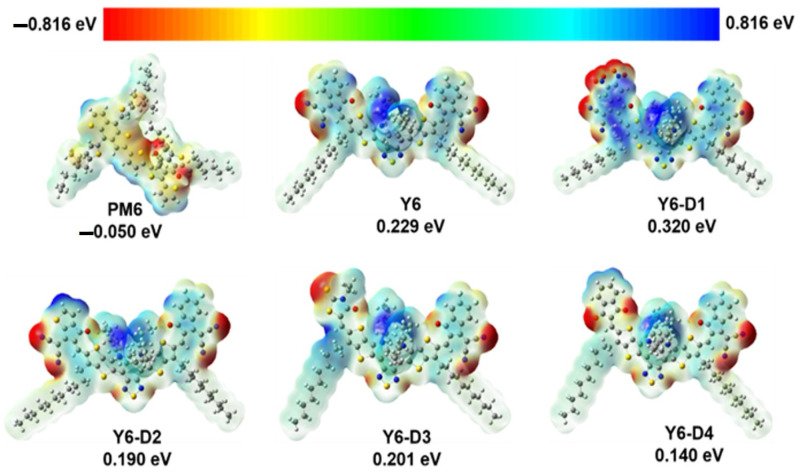
Electrostatic potential (ESP) maps of PM6, Y6, Y6-D1, Y6-D2, Y6-D3, and Y6-D4 calculated at B3LYP/6-31G(d)/polarizable continuum model (PCM) theory (ε = 3.0) level. The average value of all atomic ESPs is the average ESP. The potential values are set from −0.816 eV (the deepest red) to 0.816 eV (the deepest blue).

The ESP maps of Y6, Y6-D1, Y6-D2, Y6-D3, Y6-D4, and the PM6 monomers are presented in Figure 2. Because of the A–DAD–A-type molecular frame of the Y6 derivatives, the negatively charged areas of these molecules are primarily located at the terminal group and the central BT unit, while the positively charged areas are present at the side alkyl chains and thiophene rings on the framework. Except for Y6-D1 (0.320 eV), the average ESPs of other asymmetric Y6 derivatives are smaller than that of Y6 (0.229 eV). A smaller average ESP is not conducive to electron acceptance and charge separation. Thus, Y6-D1 with high ESP is suitable for acceptor molecules in OSCs from this perspective.

### 2.2. Molecular Packing 

**Figure 3:**
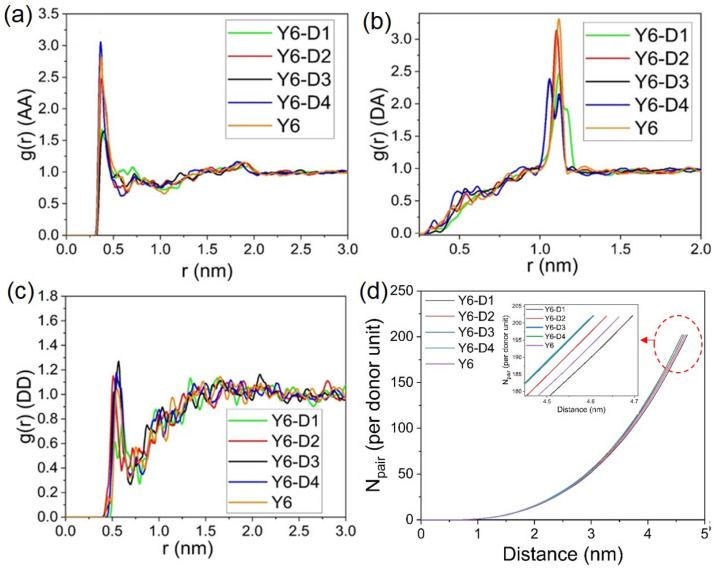
(**a**) Radial distribution function analysis of molecular acceptor unit (A–A); (**b**) donor and acceptor units (D–A); (**c**) donor unit (D–D) (without alkyl chains) in amorphous thin films; (**d**) coordination number analysis of donor unit (D–D). The curves in the red circle are magnified.

Radial distribution function (RDF) analysis of double acceptor units (A–A), donor–acceptor units (D–A), double donor units (D–D), and coordination number analysis of donor units (D–D) are depicted in Figure 3. For clarity of observations, we remove the alkyl chains of Y6 and Y6 derivatives. The RDF value of D–D (<1.4) is considerably smaller than that of A–A (<3.1) and D–A (<3.3), revealing that the D–D stacking is not dominant in the amorphous state. This finding is consistent with the results of an MD simulation of a previous “A–D–A–D–A” non-fullerene electron acceptor Y6, which is dominated by the A–A stacking of terminal groups [10]. In the main A–A stacking RDF analysis, no considerable difference among the five molecules is observed, and the stacking distance is ~0.4 nm. Thus, the optimal stacking distance between molecules is considered to be ~0.4 nm. The peaks in RDF analysis of D–A stacking represent the intramolecular ones. Moreover, the results of the coordination number analysis of D–D reveal that the N_pairs_ of Y6-D3 and Y6-D4 are higher than those of other molecules, indicating that Y6-D3 and Y6-D4 are more likely to form molecular pairs compared to Y6, Y6-D1, and Y6-D2.

**Figure 4:**
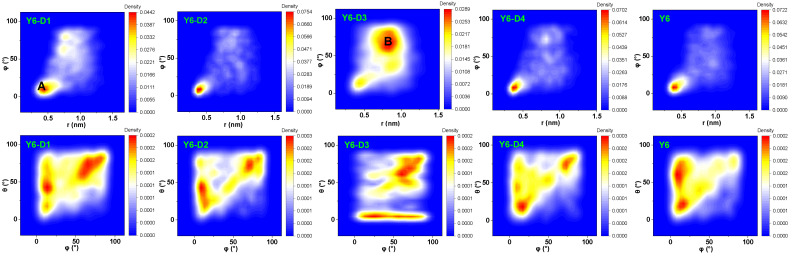
r–φ and θ–φ density contour plots of the final main A–A stacking of the five molecular dynamics simulation boxes. r: the center-of-mass distance between the terminal groups; φ: the angle between the normal directions of the terminal planes; θ: the angle between the long axes of the terminals. A: face-on configuration; B: edge-on configuration.

**Figure 5:**
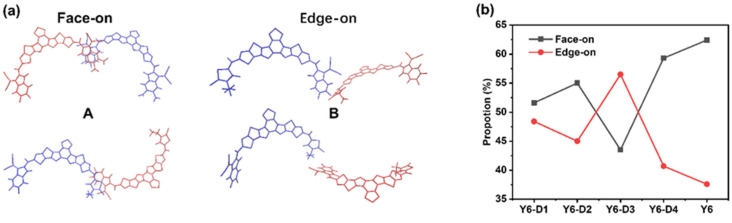
(**a**) Illustration of the two representative configurations of A–A stacking. Note that the side alkyl chains are omitted for clarity; (**b**) proportion of face-on and edge-on dimer configurations for A–A stacking in the final MD box. A: face-on configuration; B: edge-on configuration.

To investigate the stacking patterns of the five molecules, the conformation of dimers formed by adjacent molecules is characterized using three parameters: the center-of-mass (COM) distance between the terminal groups (r), the angle between the normal directions of the terminal planes (φ), and the angle between the long axes of the terminals (θ), as depicted in SI Figure S1. The RDF analysis reveals that the molecules are primarily dominated by the A–A stacking of the terminal groups, and the stacking is ~0.4 nm; however, the specific stacking mode of the end groups is unknown. Thus, we analyze the morphological stacking between the end groups, as shown in Figure 4. Careful analyses of the r, φ, and θ between the A parts of the molecular pairs in the final simulation box (a bimolecular system in which the shortest distance between atoms of two A parts is <0.5 nm is defined as a pair) indicated two representative dimer configurations. The structure and classification of the conformers A and B are shown in Figure 5a. The typical conformation A (r of ~0.4 nm, φ < 45°) exhibits the strongest parallel π–π stacking between A parts. Configuration B exhibits an r of ~0.75 nm and φ > 45°. Configuration B is mainly from Y6-D3 due to the asymmetric character of Y6-D3 as IC-2F (A1) and 3-ethyl-2-thioxothiazolidin-4-one (A3). The monocyclic structure of A3 is not favorable for parallel π–π stacking. Thus, depending on the value of φ, dimers can be roughly classified into face-on (φ < 45°) and edge-on (φ > 45°) via A–A stacking. Therefore, we identify the molecular pairs of class A as face-on configurations and B as edge-on configurations. Face-on or edge-on configurations in this research differ from those in the experimental studies. The latter describes the orientation (parallel or perpendicular) of molecular backbones to the substrate [11]. Here, the major orientation is the partial stacking between neighboring molecules. Face-on means that two adjacent molecules have face-to-face orientations of backbones (the dihedral angle between the planes of two backbones is generally smaller than 45°), and edge-on denotes that two adjacent molecules own edge-to-edge orientations (the dihedral angle between the planes of two backbones is generally not less than 45°).

The proportions of face-on and edge-on configurations are shown in Figure 5b. As expected, Y6-D3 exhibits the lowest percentage (43.5%) of face-on configurations. In comparison, the proportion of face-on configurations for the A–A stacking of Y6-D1, Y6-D2, Y6-D4, and Y6 is 51.6%, 55.0%, 59.3%, and 62.4%, respectively. Thus, for asymmetric molecules, the trend of the face-on ratio is Y6-D4 > Y6-D2 > Y6-D1 > Y6-D3. Thus, the use of oxygen groups in place of cyano groups and the elimination of fluorine substitutions may provide a better method for asymmetric modification. In addition, the use of a thiophene ring instead of a benzene ring in the end group has the potential to increase the charge transfer rate.

### 2.3. Reorganization Energy and Charge Carrier Mobility

**Figure 6:**
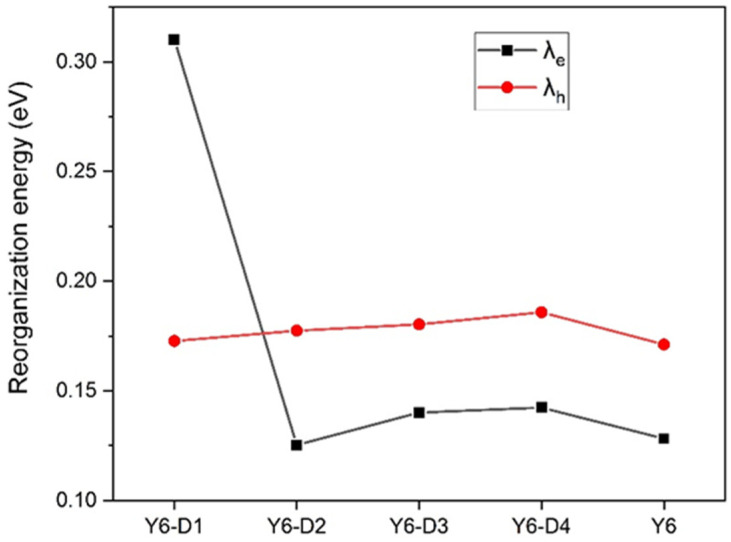
Electronic (λ_e_) and hole (λ_h_) inner reorganization energies of Y6, Y6-D1, Y6-D2, Y6-D3, and Y6-D4 with B3LYP/6-31G(d) theory level.

The inner reorganization energies of Y6, Y6-D1, Y6-D2, Y6-D3, and Y6-D4 are shown in Figure 6. The internal reorganization energy characterizes the degree of geometric distortion of molecules when transferring charges. The smaller the internal reorganization energy, the easier the transfer of charges, which is beneficial for carrier transport. Except for Y6-D1 substituted by one IC-2NO_2_ terminal group with the strongest electron-withdrawing ability, the internal reorganization energy of the electron transport is generally lower than that of hole transport. Thus, Y6, Y6-D2, Y6-D3, and Y6-D4 are primarily electron transport oriented. The hole inner reorganization energy of Y6-D1 is not significantly different from that of other derivatives, so IC-NO_2_ mainly affects the electron inner reorganization energy. Due to the presence of IC-NO_2_ at one end, electron-withdrawing ability is too strong. After electron transfer, the energy required to change the internal structure is high, so the electron inner reorganization energy becomes large, and a reversal occurs. Electron transport may transit to hole transport for the Y6 derivative by connecting a strong electron-withdrawing terminal. Y6-D2 exhibits the smallest electronic inner reorganization energy (λ_e_) value, suggesting the easiest electron transfer. 

**Figure 7:**
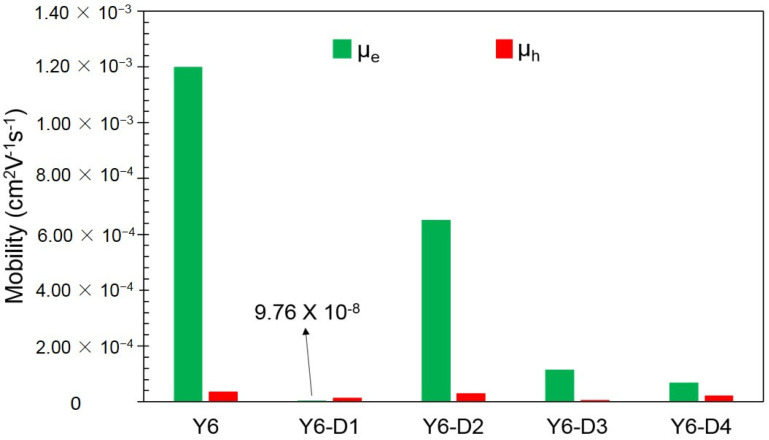
Electron (μ_e_) and hole (μ_h_) mobilities of Y6, Y6-D1, Y6-D2, Y6-D3, and Y6-D4 obtained using molecular dynamics and kinetic Monte Carlo simulations.

The electron mobility (μ_e_) and hole mobility (μ_h_) of Y6, Y6-D1, Y6-D2, Y6-D3, and Y6-D4 are shown in Figure 7. Except for that of Y6-D1, replaced by the strongest electron-withdrawing IC-2NO_2_ terminal group, the hole mobilities of the other three asymmetric Y6 derivatives are smaller than the electron mobilities. This confirms that these asymmetric molecules are mainly electron-transporting and can be used as acceptors. Figure 7 shows that the simulated electron mobility of pure Y6 film is 12.0 × 10^−4^ cm^2^ V^−1^ s^−1^, which is close to the results of an experimental blend film (PM6:Y6, μ_e_ = 5.2 × 10^−4^ cm^2^ V^−1^ s^−1^) [12], verifying the accuracy of our calculation method. Y6-D2 exhibits an electron mobility of 6.5 × 10^−4^ cm^2^ V^−1^ s^−1^, which is close to that of Y6. 

### 2.4. Binding Energies of PM6/Asymmetric Y6 Derivative Bimolecular Systems

An optimized PM6/Y6 bimolecular system was calculated by our group in previous work [13], and the configuration is shown in Figure S2. The acceptor (A) part of Y6 stacks to the A group of PM6 to obtain a stable configuration. Thus, for each asymmetric terminal-based Y6 derivative, there are two bimolecular systems. The A1 part of the asymmetric Y6 derivative stacks to the A group of PM6 to form one bimolecular system (for example, PM6/Y6-D1-1), and the A3 part of the asymmetric Y6 derivative stacks to the A group of PM6 to form another bimolecular system (for example PM6/Y6-D1-2). 

**Figure 8:**
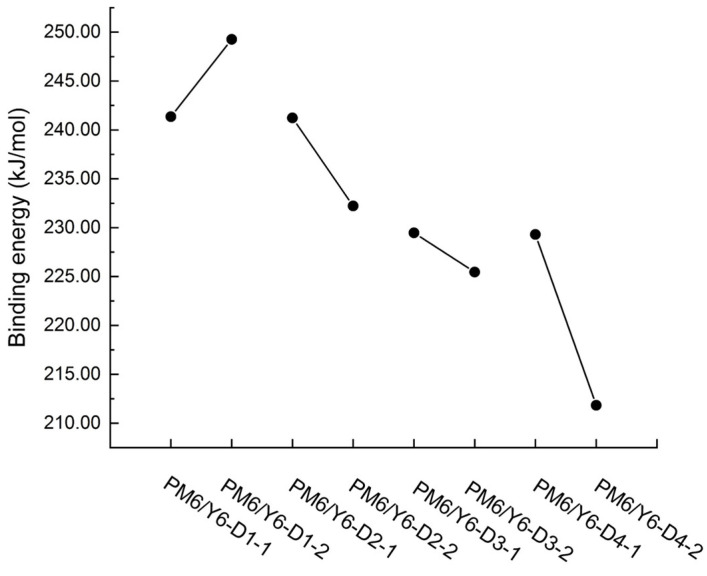
Binding energies of PM6/Y6-D1-1, PM6/Y6-D1-2, PM6/Y6-D2-1, PM6/Y6-D2-2, PM6/Y6-D3-1, PM6/Y6-D3-2, PM6/Y6-D4-1, and PM6/Y6-D4-2 bimolecular systems.

Binding energy is an important parameter for evaluating the binding tightness between two bodies. The larger the binding energy, the more stable the structure. The binding energies of the different bimolecular systems of PM6/Y6-D1, PM6/Y6-D2, PM6/Y6-D3, and PM6/Y6-D4 are calculated using Equation (4), and the results are shown in Figure 8. PM6/Y6-D1-2 (241.262 kJ/mol), PM6/Y6-D2-1 (241.232 kJ/mol), PM6/Y6-D3-1 (229.483 kJ/mol), and PM6/Y6-D4-1 (229.310 kJ/mol) have larger binding energies, indicating a stable structure. The stable bimolecular systems were selected to calculate the HOMO, LUMO, energy gap, and UV–Vis spectra.

### 2.5. FMOs and Gap Energies of PM6/Asymmetric Y6 Derivative Bimolecular Systems

**Figure 9:**
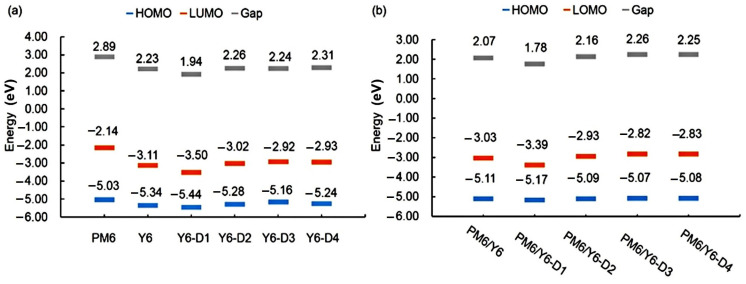
HOMO and LUMO energy levels and energy gaps of the (**a**) PM6, Y6, Y6-D1, Y6-D2, Y6-D3, and Y6-D4 monomers; (**b**) PM6/Y6, PM6/Y6-D1, PM6/Y6-D2, PM6/Y6-D3, and PM6/Y6-D4 bimolecular systems calculated with a tuned ωB97X/6-31+G(d)/PCM theory level.

**Figure 10:**
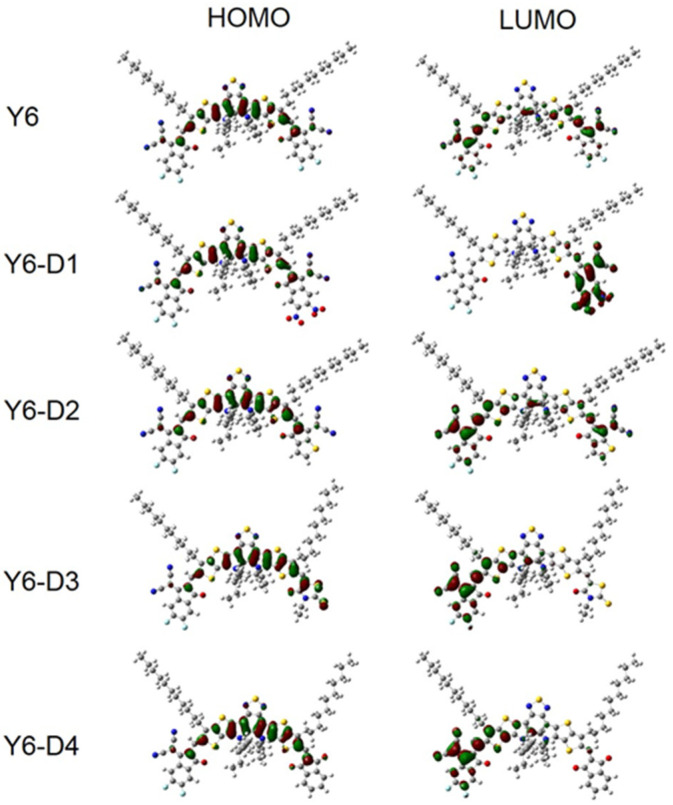
The frontier molecular orbitals of Y6, Y6-D1, Y6-D2, Y6-D3, and Y6-D4 obtained with tuned ωB97X/6-31+G(d)/PCM theory level.

The HOMO and LUMO energies and energy gaps of the PM6, Y6, Y6-D1, Y6-D2, Y6-D3, and Y6-D4 monomers as well as the bimolecular systems are shown in Figure 9. Figure 9a indicates that the energy levels of PM6 match those of Y6 derivatives with asymmetric terminal groups. Among these asymmetric Y6 derivatives, Y6-D1 exhibits the smallest energy gap. The electron-withdrawing –NO_2_ group reduces the HOMO and LUMO energy levels and the energy gap of this molecule. The HOMO and LUMO energy levels of Y6-D2 and Y6-D3 are considerably increased, and the energy gap is not considerably different from that of Y6. Compared to Y6, the HOMO and LUMO energies of Y6-D4 considerably increased, and Y6-D4 exhibited the largest energy gap. The substitution of 1H-indene-1,3(2H)-dione increases the energy gap. Compared to Y6, except for Y6-D1, the increased LUMO levels of Y6-D2, Y6-D3, and Y6-D4 may be the reason for the higher V_OC_ of OSCs based on them rather than a Y6 acceptor [14]. As shown in Figure 9b, compared to the PM6/Y6 bimolecular system, the HOMO and LUMO energy levels and the energy gaps of the asymmetric Y6-D1 (substituted by the strongest electron-withdrawing IC-2NO_2_) and PM6 bimolecular system decrease. However, the HOMO and LUMO energy levels and the energy gaps of the other bimolecular systems considerably increase. We also draw the frontier molecular orbital images of Y6 and Y6 asymmetric derivatives, as shown in Figure 10. Clearly, there is no significant difference in the electron distribution on the HOMOs of Y6 and Y6-D1, Y6-D2, Y6-D3, and Y6-D4. However, there are noticeable differences in the distribution of electrons on LUMOs. For symmetric Y6, LUMO is mainly on two same terminals (IC-2F). Compared to Y6, the LUMO of asymmetric Y6-D1 is only on one terminal group IC-NO_2_, and not on another terminal group IC-2F, because the electron withdrawing ability of the IC-2NO_2_ group is greater than that of IC-2F. Similarly, the LUMOs of Y6-D3 and Y6-D4 are mainly in IC-2F group.

### 2.6. UV–Vis Spectra of Bimolecular Systems

**Figure 11:**
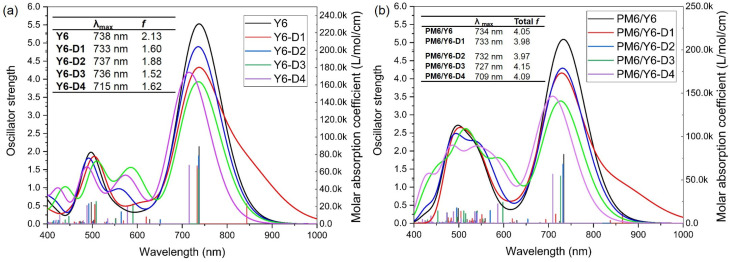
Simulated UV–vis absorption spectra of the (**a**) five acceptors (monomers) and (**b**) five bimolecular systems. “λ_max_” and “*f*” in (**a**) are the wavelength and corresponding oscillator strength of the main absorption peaks, respectively, and “Total *f*” in (**b**) denotes the total oscillator strength in the range of 400–1000 nm. All TDDFT results are obtained with a tuned ωB97X/6-31G(d)/PCM theory level, and the half-peak width of the simulated UV–vis absorption spectra was set at 0.25 eV.

As mentioned, –NO_2_ down-shifts the optical gap and broadens the absorption spectrum of fused-ring electron acceptors (FREAs). Compared to the Y6 monomer (Figure 11a), the main peak of the absorption spectrum of the Y6-D1 monomer is broadened. The main peaks of Y6-D1, Y6-D2, and Y6-D3 are almost at the same wavelength as Y6, and Y6-D4 is blue-shifted. However, comparing the donor/acceptor bimolecular systems (Figure 11b) to the PM6/Y6 prototype, the main peaks of PM6/Y6-D1 and PM6/Y6-D2 are almost at the same wavelength as the PM6/Y6 prototype, whereas PM6/Y6-D3 and PM6/Y6-D4 are blue-shifted. PM6/Y6-D1 and PM6/Y6-D2 exhibit slightly smaller total oscillator strengths than those of the PM6/Y6 prototype, whereas PM6/Y6-D3 and PM6/Y6-D4 maintain slightly higher total oscillator strengths due to the suitable electron-withdrawing capacities of the terminal groups of Y6-D3 and Y6-D4. This demonstrates the greater light absorption of PM6/Y6-D3 and PM6/Y6-D4.

## 3. Computational Details

To simulate a solid-state environment, an integral equation formalism variant polarizable continuum model (PCM) [15] was used for all calculations. Previous studies have found that the relative dielectric constants (ε) of a common NFA and D–A copolymer donor range between 2.65 and 3.26; therefore, ε was set at 3.0 in this study [16,17]. Geometric optimizations and frequency calculations of all ground-state monomers were carried out at the B3LYP/6-31G(d) theoretical level [18], which has been proven to effectively reproduce the experimental structure [19]. The FMO and excited state energy calculations were based on the long-range correction (LRC) density functional tunable ωB97X [20] with 6-31+G(d) and 6-31G(d) basis sets respectively, which has proven to be accurate for Y6 and its derivatives. All quantum-chemical calculations were conducted using the Gaussian 09 Rev E.01 software package [21]. 

For the LRC functional tunable ωB97X with a range-separated interval separation parameter ω, the ωs of the acceptor and donor molecules were optimized with the PCM using the following equations.

(1) $$J^{2}\left(\omega \right)=J_{N}^{2}\left(\omega \right)+J_{N+1}^{2}\left(\omega \right)$$

(2) $$J_{N}^{2}\left(\omega \right)=[\varepsilon_{HOMO}^{\omega }\left(N\right)+E^{\omega }\left(N-1\right)-E^{\omega }(N){]}^{2}$$

(3) $$J_{N+1}^{2}\left(\omega \right)=[\varepsilon_{HOMO}^{\omega }\left(N+1\right)+E^{\omega }\left(N\right)-E^{\omega }(N+1){]}^{2}$$

where $E^{\omega }\left(N\right)$ represents the energy of a neutral system, $\varepsilon_{HOMO}^{\omega }\left(N\right)$ represents the energy of the HOMO of the neutral system, $E^{\omega }(N+1)$ represents the energy of the anion, and $E^{\omega }\left(N-1\right)$ represents the energy of the cation. The optimized ωs for Y6, Y6-D1, Y6-D2, Y6-D3, Y6-D4, and PM6 are 0.016, 0.014, 0.017, 0.018, 0.018, and 0.014 (Bohr−a), respectively.

Multiwfn 3.8 software was adopted to simulate the absorption spectra, which were output as TDDFT calculations [22]. The full width at the half maximum of the absorption peak was set at 0.25 eV. 

The binding energy (*E_binding_*), which directly affects interfacial binding interactions, was considered using the following equation [23]:

(4) $$E_{binding}=E_{A/D}-E_{A}-E_{D}+E_{BSSE}$$

where $E_{A/D}$ represents the total energy of the bimolecular donor and acceptor, $E_{A}$ represents the energy of the acceptor, $E_{D}$ denotes the energy of the donor, and $E_{BSSE}$ indicates the energy of the basis set superposition error. 

Because the solvent molecules do not undergo large deflections in a thin-film environment, the external reorganization energy is small and negligible. Only the inner reorganization energy was considered in this work. The inner reorganization energy for electron transport was calculated using the following equation:

(5) $$\begin{array}{c} \;\lambda_{i,e}=\left[E^{0}\left(A^{-}\right)-E^{0}\left(A^{0}\right)\right]+\left[E^{-}\left(A^{0}\right)-E^{-}\left(A^{-}\right)\right] \end{array}$$

where $\lambda_{i,\;e}$ is the inner reorganization energy during electron transfer, $E^{0}\left(A^{-}\right)\;means$ the total energy of the neutral acceptor in the optimized geometry of the anion, $E^{0}\left(A^{0}\right)$ denotes the total energy of the neutral acceptor in the optimized geometry of the neutral molecule, $E^{-}\left(A^{0}\right)$ represents the energy of the negative acceptor in the optimized geometry of the neutral molecule, and $E^{-}\left(A^{-}\right)$ means the energy of the negative acceptor in the optimized geometry of the anion. 

Similarly, the inner reorganization energy of the hole transport was simulated using the following equation:

(6) $$\begin{array}{c} \;\lambda_{i,h}=\left[E^{0}\left(D^{+}\right)-E^{0}\left(D^{0}\right)\right]+\left[E^{+}\left(D^{0}\right)-E^{+}\left(D^{+}\right)\right] \end{array}$$

where $E^{+}\left(D^{0}\right)-E^{+}\left(D^{+}\right)$ means the energy required for the geometric structure of the positive ions to relax into the geometric structure of the neutral molecules in the positive ion state, and $E^{0}\left(D^{+}\right)-E^{0}\left(D^{0}\right)$ represents the energy required for the geometric structure of the neutral molecules to relax into the geometric structure of the positive ions in the neutral electronic state.

All molecular dynamics simulations were calculated using the GROMACS 5.1.3 software package [24], and GaussView 5.0.8 was used for molecular modeling [25]. ESP was calculated using Gaussian 09 Rev E.01, and Multiwfn 3.8 [26,27] was used to simulate the restricted ESP charge [28,29] based on the ESP data. ACPYPE tools [30] were used to generate parameters and topology based on the general AMBER force field [31]. The torsional potential parameters between the alkyl chain and thiophene ring, the alkyl chain and pyrrole ring, and thiophene and the π-bridge are simulated by DFT calculation of B3LYP/6-31G(d), as shown in Figure S3. 

The molecular dynamics calculation steps, as shown in Figure S4 [32,33], were as follows: (1) Packmol software (Version 17.039) [34] was used to randomly place 400 molecules in a 40 × 40 × 40 nm^3^ box to randomly orientate the molecules, (2) 100,000 energy minimization steps using the steepest descent method were conducted to eliminate structural deformation and unnecessary force, (3) 5 ns compression at 600 K and 100 bar was used to narrow the distance between the molecules, (4) annealing for 10 ns at 600 K and 1 bar and cooling to 300 K in 3 ns were carried out to simulate the experimental thermal annealing process, and (5) relaxation for 20 ns at 300 K and 1 bar to obtain the equilibrium system. Periodic boundary conditions were used for all the MD calculations, with a time step of 1 fs. The cutoff for short-range interactions, such as the Coulomb force, was set at 1.2 nm. Throughout the entire simulation, a V-rescale thermostat [35] and a Berendsen barostat [36] were used under NPT, except for the last 10 ns calculation for Step 5, where we used a Nosé–Hoover thermostat [37,38] and a Parrinello–Rahman barostat [39] to obtain a better equilibrium system. The last 0.5 ns of relaxation was used to analyze the statistical RDFs.

We chose the hopping model [40] to obtain the charge mobility because the recombination energy is usually larger than the electronic coupling in our simulated system. The carrier mobility was simulated using the Einstein–Smoluchowski formula [41] as follows:

(7) $$\begin{array}{c} \;\mu =\frac{eD}{k_{B}T}\;\; \end{array}$$

where μ represents the carrier mobility, e represents the amount of electronic charge, $k_{B}$ represents the Boltzmann constant, D represents the diffusion coefficient, and T represents the absolute temperature. The diffusion coefficient D was simulated using the following formula:

(8) $$\begin{array}{c} D=\frac{1}{2n}\underset{t\to \infty }{\lim}\frac{{r\left(t\right)}^{2}}{t} \end{array}$$

where n represents the dimension of the system (n = 1–3), t represents the diffusion time, and ${r\left(t\right)}^{2}$ represents the mean square displacement. A kinetic Monte Carlo (KMC) simulation [42] was run to mimic the charge hopping process in the amorphous films, which were obtained from the previous CMD simulations. For all molecular pairs in the KMC simulations, we treated the COM of each molecule as the hopping point. Initially, one molecule (*i*) was randomly selected in the CMD simulation box. Then, an electron or hole transfers between molecule *i* and its adjacent molecule (*j*). The probability of this charge hopping is defined as $P_{ij}=\sum_{n}k_{ij}$, in which *k_ij_* is the hopping rate from molecule *i* to *j*.

The charge hopping rates between asymmetric Y6 derivatives were simulated using semiclassical Marcus theory [43] as follows:

(9) $$\begin{array}{c} k_{ij}=\frac{{V_{ij}}^{2}}{\hbar }\sqrt{\frac{\pi }{\lambda k_{B}T}}exp\left[-\frac{{\left(\Delta G_{ij}+\lambda \right)}^{2}}{4\lambda k_{B}T}\right] \end{array}$$

where ℏ represents the simplified Planck constant, $k_{B}$ represents the Boltzmann constant; λ represents the reorganization energy calculated using B3LYP/6-31G(d), T represents the temperature (set at 300 K), $V_{ij}$ represents the transfer integral between the HOMOs of two interacting molecules derived using the Zerner intermediate neglected differential overlap method [44], and $\Delta G_{ij}$ is the free energy difference of the charge transfer between two molecules (set as zero).

## 4. Conclusions

Using DFT, TDDFT, and MD calculations, we studied the photovoltaic properties of Y6 derivatives with different asymmetric terminal groups. RDF data show that A–A stacking is dominant for Y6 and four asymmetric Y6 derivatives. For asymmetric Y6 derivatives, Y6-D3 has the lowest percentage (43.5%) of face-on configurations of A-A stacking. In contrast, the percentages of Y6-D1, Y6-D2, and Y6-D4 are 51.6%, 55.0%, and 59.3%, respectively. The Y6-D4 face-on ratio of A–A stacking is highest, followed by Y6-D2, Y6-D1, and Y6-D3. The results of electron- and hole-mobility calculations indicate that, except for Y6-D1, the other three asymmetric molecules are mainly electron-transporting and can be used as acceptors. The higher LUMO levels of Y6-D2, Y6-D3, and Y6-D4 may demonstrate the higher V_OC_ of OSCs based on Y6-D2, Y6-D3, and Y6-D4 compared to the Y6 acceptor. UV–Vis spectra show that PM6/Y6-D3 and PM6/Y6-D4 have higher total oscillator strengths than that of PM6/Y6, indicating that PM6/Y6-D3 and PM6/Y6-D4 have better light absorption because of the suitable electron-withdrawing ability of the terminal groups of Y6-D3 and Y6-D4. Based on these results, Y6-D3 and Y6-D4 may be good acceptors.

## Data Availability

Not applicable.

## Supplementary Materials

The following supporting information can be downloaded at: https://www.mdpi.com/article/10.3390/ijms241914753/s1. The following references were cited in supplementary materials [13,32,33,45,46,47].
