# Supplementary material for: Enhanced Photovoltaic Properties of Y6 Derivatives with Asymmetric Terminal Groups: A Theoretical Insight
Source: Int J Mol Sci. 2023 Sep 29;24(19):14753. doi: 10.3390/ijms241914753 (PMC10572497; doi:10.3390/ijms241914753)
Supplement: Supplementary file 1 [file ijms-24-14753-s001.zip › ijms-2590973-supplementary.pdf]

SI

Enhanced photovoltaic properties of Y6 derivatives with asymmetric terminal groups: A theoretical insight

Yunjie Xiang[1,2], Zhijun Cao[1,2], Xiaolu Zhang[1,2], Zhuo Zou[3,*] and Shaohui Zheng[1,2,*]

[1]School of Materials and Energy, Southwest University, Chongqing, China

[2]Chongqing Key Laboratory for Advanced Materials and Technologies of Clean Energies, Southwest University, Chongqing, China

[3]School of Materials Science and Engineering, Suzhou University of Science and Technology, Jiangsu, China

Corresponding author: zouzhuo@usts.edu.cn; shaohuizheng@swu.edu.cn

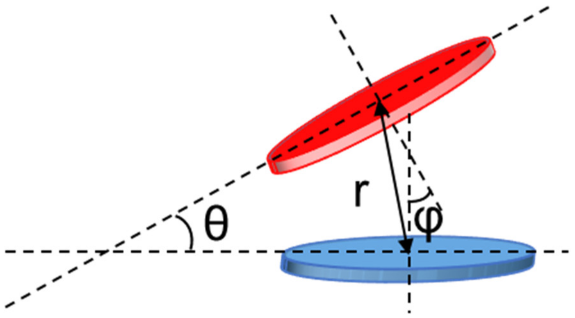

Figure S1. Three geometric parameters of the dimer configuration: the centroid distance between the terminal groups (r), the angle between the normal directions of the terminal planes (φ), and the angle between the long axes of the terminals (θ).

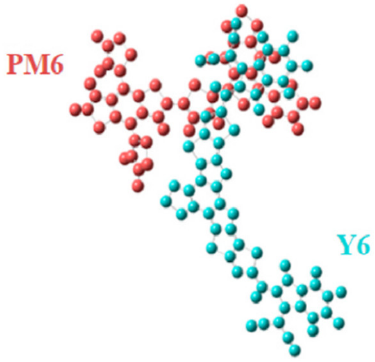

Figure S2. Optimized PM6 (red) and Y6 (blue) bi-molecular configuration [1]. For clarity, the alkyl chains are removed which are retained in all calculations.

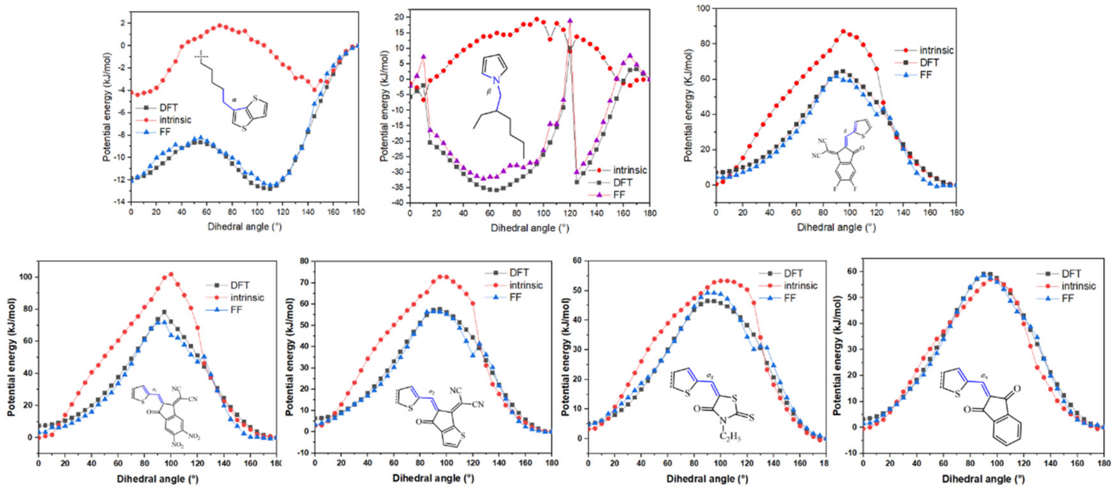

Figure S3. The total potential energy simulated by DFT and GAFF, and the fitting intrinsic potential between the alkyl chain and thiophene, alkyl chain and pyridine ring, and thiophene and terminal group.

The torsional potential parameters of key dihedral-angles are fitted by DFT simulation, and the steps

are as follows.[2-6]

1. Extract the part of molecule to be fitted, and perform geometric optimization through B3LYP/6-31G (d).

2. Scan the restricted flexible potential energy surface of the dihedral-angle to be fitted by Gaussian, every 5° from 0° to 180°, and obtain the DFT energy for each step.

3. GROMACS is used to simulate the single point energy of each step in the scanning process in step 2, and get the MM energy of the dihedral angle to be fitted.

4. Subtract the MM energy obtained in the third step from the DFT energy got in the second step to get the intrinsic energy of the dihedral angle to be fitted.

5. The intrinsic energy is fitted by Rychart Bellemans formula to get the torsional potential parameters.

6. Insert the obtained torsional potential parameters into the force field parameter file to get the force field energy and compare it with the DFT energy curve. If the coincidence degree is high, the fitting is successful.

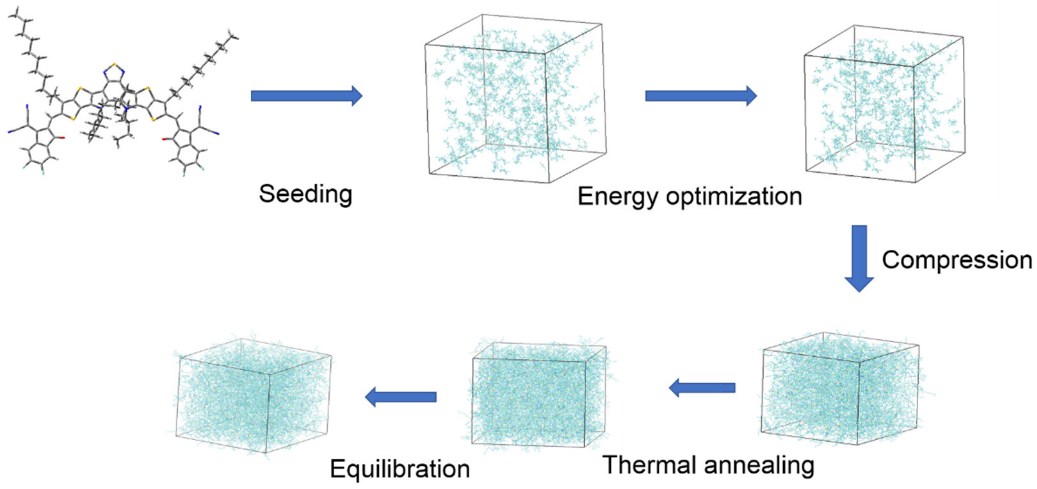

Figure S4. Molecular dynamics simulation process of asymmetric Y6 derivatives

[1] Qiu W, and Zheng S. Effects of functionalization of Y6 end-groups with electron-withdrawing groups on the photovoltaic properties at the donor-acceptor interfaces of PM6/Y6 OSCs: A theoretical insight. *Organic Electronics,* **2021**, *96*, 106235.

[2] Guilbert A A Y, Frost J M, Agostinelli T, et al. Influence of Bridging Atom and Side Chains on the Structure and Crystallinity of Cyclopentadithiophene–Benzothiadiazole Polymers[J]. *Chemistry of Materials*, **2014**, 26 (2): 1226-1233.

[3] Han G C, Guo Y, Duan R H, et al. Importance of side-chain anchoring atoms on electron donor/fullerene interfaces for high-performance organic solar cells[J]. *Journal of Materials Chemistry A*, **2017**, 5 (19): 9316-9321.

[4] Moreno M, Casalegno M, Raos G, et al. Molecular Modeling of Crystalline Alkylthiophene Oligomers and Polymers[J]. *Journal of Physical Chemistry B*, **2010**, 114 (4): 1591-1602.

[5] Poelking C, Andrienko D. Effect of Polymorphism, Regioregularity and Paracrystallinity on Charge Transport in Poly(3-hexylthiophene) [P3HT] Nanofibers[J]. *Macromolecules*, **2013**, 46 (22): 8941-8956.

[6] Xu C L, Yao C, Zheng S H. Effects of lateral-chain thiophene fluorination on morphology and charge transport of BDT-T based small molecule donors: a study with multiscale simulations[J]. *Journal of Materials Chemistry C*, **2021**, 9 (41): 14637-14647.
